# Leveraging artificial intelligence in disaster management: A comprehensive bibliometric review

**DOI:** 10.4102/jamba.v17i1.1776

**Published:** 2025-04-07

**Authors:** Arief Wibowo, Ikhwan Amri, Asep Surahmat, Rusdah Rusdah

**Affiliations:** 1 Department of Computer Science, Faculty of Information Technology, Universitas Budi Luhur, Jakarta, Indonesia; 2 Center for Disaster Studies, Universitas Gadjah Mada, Yogyakarta, Indonesia; 3 Department of Information System, Faculty of Technology and Design, Universitas Utpadaka Swastika, Tangerang, Indonesia

**Keywords:** artificial intelligence, disaster management, natural hazard, bibliometric analysis, Scopus

## Abstract

The advancement of artificial intelligence (AI) technology presents promising opportunities to improve disaster management’s effectiveness and efficiency, particularly with the rising risk of natural hazards globally. This study used the Scopus database to offer a bibliometric review of AI applications in disaster management. Publications were chosen based on research scope (natural hazards), source type (journals and conference proceedings), document type (articles, conference papers and reviews) and language (English). VOSviewer and Biblioshiny were utilised to analyse trends and scientific mapping from 848 publications. The finding shows a rapid increase in AI studies for disaster management, with an annual growth rate of 15.61%. The leading source was the International Archives of the Photogrammetry, Remote Sensing and Spatial Information Sciences – ISPRS Archives. Amir Mosavi was the most prolific author, with 10 documents. The analysis reveals that China was the most productive country, while the United States was the most cited. Six research clusters were identified through keyword network mapping: (1) disaster monitoring and prediction using IoT networks, (2) AI-based geospatial technology for risk management, (3) decision support systems for disaster emergency management, (4) social media analysis for emergency response, (5) machine learning algorithms for disaster risk reduction, and (6) big data and deep learning for disaster management.

**Contribution:**

This research contributes by mapping the application of AI technology in disaster management based on peer-reviewed literature. This helps identify major developments, research hotspots, and gaps.

## Introduction

Artificial intelligence (AI) enables machines to perform tasks that typically require human intelligence, such as logical reasoning, learning and problem-solving, through algorithms and machine learning (ML) technologies (Morandín-Ahuerma 2022). Artificial intelligence has evolved from a basic idea to a sophisticated system capable of achieving goals via adaptable learning (Haenlein & Kaplan 2019). With its vast potential, AI can transform industries and society. However, ongoing research and addressing implementation challenges are essential for successful integration and future impact (Dwivedi et al. 2021).

As natural hazards become more frequent and intense, AI technology plays a vital role in processing various data types to enhance disaster understanding, improve forecasting and support humanitarian relief (Pang 2022). Artificial intelligence-based models can accurately detect early disaster signs, helping emergency managers take proactive measures to reduce impacts (Sharma et al. 2022). Artificial intelligence can also quickly assess disaster damage (Takhtkeshha, Mohammadzadeh & Salehi 2023). Thus, AI development positively impacts disaster management across all phases, from pre-disaster to post-disaster (Tan et al. 2021). However, a comprehensive examination of publication trends and thematic developments in this field has not been extensively conducted.

Bibliometric studies are vital for mapping research developments. Recent reviews of AI applications cover fields such as medicine (Frasca et al. 2024), education (Kavitha et al. 2024), tourism and hospitality (González-Mendes, González-Sanchez & Alonso-Muñoz 2024), and healthcare (Shah et al. 2024). However, there is a notable gap in bibliometric studies on AI in disaster management. In fact, AI innovation has been rapidly increasing in recent years, positioning it as an exceptionally fitting tool to tackle the complex and diverse challenges of contemporary disaster management (Sun, Bocchini & Davison 2020). The application of AI in disaster management may encounter unique challenges, such as the need for real-time decision-making or managing large-scale humanitarian crises, setting it apart from its practices in other fields (Abid et al. 2021).

A bibliometric review is crucial for gaining a comprehensive understanding of research trends through quantitative evidence (Achadi et al. 2024). This type of analysis enables the exploration of vast amounts of scientific data, uncovering emerging areas and tracing the evolution of specific fields (Donthu et al. 2021). Additionally, research profiling based on bibliometrics assists researchers in identifying key hotspots and gaps for further exploration (Zupic & Čater 2014).

This study aims to perform a bibliometric analysis of AI research in disaster management, particularly concerning natural hazards. This article seeks to answer the following research questions: (1) How has the literature on AI for disaster management evolved? (2) Which sources, authors, affiliations and countries are most active in this field? (3) Which articles and countries contribute most to citations? (4) What are the trends in using author keywords in this area?

## Research methods and design

A literature search on AI technology in disaster management was conducted using the Scopus database, known for its high-quality, peer-reviewed scientific publications across various disciplines (Kähler 2010). Obtaining publication data from Scopus is straightforward with simple or advanced queries. It provides comprehensive information, including citation details, bibliographical data, abstracts, keywords and funding information, which can be exported in various formats.

**FIGURE 1:**
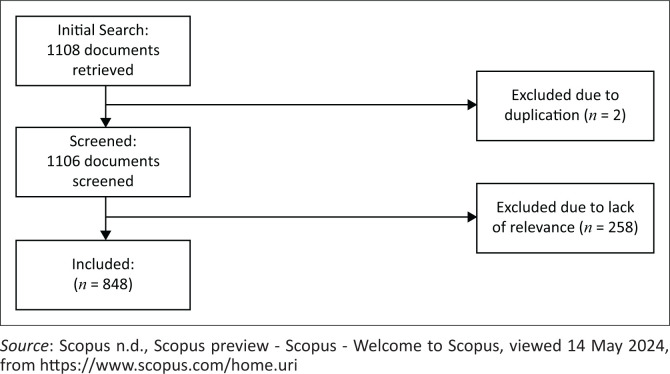
Document selection process.

Document extraction was conducted on 14 May 2024, from the Scopus database using the following search formula: (TITLE-ABS-KEY(‘artificial intelligence’) AND TITLE-ABS-KEY(‘natural disaster’ OR ‘natural hazard’ OR ‘disaster management’ OR ‘disaster emergenc*’ OR ‘disaster risk reduction’ OR ‘disaster risk management’)) AND (LIMIT-TO(SRCTYPE, ‘j’) OR LIMIT-TO(SRCTYPE, ‘p’)) AND (LIMIT-TO(DOCTYPE, ‘cp’) OR LIMIT-TO(DOCTYPE, ‘ar’) OR LIMIT-TO(DOCTYPE, ‘re’)) AND (LIMIT-TO(LANGUAGE, ‘English’)). This study focused on natural hazards, excluding disease outbreaks, technological hazards and social hazards. By focusing on this area, the findings will be more pertinent to researchers and practitioners in the field, as natural hazards often pose distinct challenges that are different from those associated with other types of hazards (Ward et al. 2020). Source types were restricted to journals and conference proceedings, while document types included articles, conference papers and reviews. Non-English documents were excluded. No limitations were applied to the publication timeframe. The complete data selection process is shown in Figure 1. The final metadata was exported as a CSV file for import into bibliometric visualisation software.

The primary software used for generating bibliometric visualisations included the R program with the *bibliometrix* package (Aria & Cuccurullo 2017) and VOSviewer. Both are popular and widely used in science mapping-based review research. Microsoft Excel was also used to create graphs for descriptive analysis. The results and discussion in this study are divided into six categories: (1) descriptive information, (2) publication growth, (3) sources, (4) authors, affiliations, and countries, (5) citations, and (6) keyword analysis.

### Ethical considerations

This study does not require ethical consideration as it does not involve human subjects, personal data, or experimental interventions.

## Results and Discussion

### Descriptive information

The general research data consisted of 848 documents extracted from the Scopus database, distributed across 551 sources, with the oldest publication dating back to 1996. The average document age was 5.7 years, indicating that most published literature was relatively recent. The total number of authors contributing to AI research in disaster management was 3056. Nearly one-third of the research (26.89%) involved international research collaboration. The majority of the document types in this research were conference papers (435; 51%), followed by articles (358; 42%) and reviews (55; 7%).

### Publication growth

Since 2010, the annual production of scientific papers has consistently reached at least 20 documents. Significant growth began in 2019 when the number of publications in a single year exceeded 50 for the first time. This trend continued steadily through 2023 and into 2024, despite the latter still being incomplete at the time of the study. According to the annual interval analysis of document growth, the increase in documents in 2022 was the highest, reaching 51, marking the first year the number of publications surpassed 100. The publication growth curve peaked in 2023 with a total of 151 documents.

### Sources

Research on AI applications for disaster management was published across various disciplines, including computer science, disaster study, earth science, geographic information science and multidisciplinary sources. This study identified the Institute of Electrical and Electronics Engineers Inc. (IEEE) as the leading publisher in this field, mainly through conference papers. However, five sources have published more than 10 documents on AI for disaster management (Table 1), with two being conference proceedings and three being journals. The International Archives of the Photogrammetry, Remote Sensing and Spatial Information Sciences – ISPRS Archives led with 22 publications. *Natural Hazards* ranked second in the journal category with 17 articles on AI for disaster management. The ACM International Conference Proceeding Series, a computer science publication outlet, ranked third. Notably, several journals managed by the Multidisciplinary Digital Publishing Institute (e.g. *Water, Remote Sensing*, and *Sustainability*) also contributed to a significant number of publications in this area.

**TABLE 1:** Top five active sources.

Source title	Source type	Publisher	Cite score (2023)	SJR (2023)	SNIP (2023)	TP
International Archives of the Photogrammetry, Remote Sensing and Spatial Information Sciences – ISPRS Archives	Conference proceeding	International Society of Photogrammetry and Remote Sensing (ISPRS)	1.7	0.28	0.38	22
*Natural Hazards*	Journal	Springer Nature	6.6	0.80	1.12	17
ACM International Conference Proceeding Series	Conference proceeding	Association for Computing Machinery (ACM)	1.5	0.25	0.23	16
*Water*	Journal	Multidisciplinary Digital Publishing Institute (MDPI)	5.8	0.72	1.00	14
*Remote Sensing*	Journal	Multidisciplinary Digital Publishing Institute (MDPI)	8.3	1.09	1.33	13

*Source:* Scopus n.d., Scopus preview - Scopus - Welcome to Scopus, viewed 14 May 2024, from https://www.scopus.com/home.uri

SJR, Scimago Journal Rank; SNIP, Source Normalised Impact per Paper; TP, total publications.

Since 2004, the International Archives of the Photogrammetry, Remote Sensing and Spatial Information Sciences – ISPRS Archives has led in the cumulative number of documents related to the development of AI publications for disaster management, with an increasing gap compared to other significant sources. *Natural Hazards* was one of the pioneers in publishing AI research for disaster management and has been active since 1998. In contrast, the ACM International Conference Proceeding Series published its first related work in 2009. Although journals affiliated with Multidisciplinary Digital Publishing Institute (MDPI) were relatively new, they rapidly increased their number of publications, demonstrating impressive achievements in a short period.

### Authors, affiliations and countries

In AI research for disaster management, several prominent authors have made significant contributions with more than five documents. The three most prolific authors who consistently published their research on AI in disaster management included Amir Mosavi from Óbuda University in Hungary, who had contributed 10 documents; Biswajeet Pradhan from the University of Technology Sydney in Australia, who had contributed eight documents; and Muhammad Imran from Hamad Bin Khalifa University in Qatar, who had contributed six documents. Each, affiliated with different institutions, played an essential role in advancing research in this field.

Table 2 highlights 10 institutions with a publication frequency exceeding 10 documents. The most productive institution was Wuhan University, which had 21 documents. In terms of countries where these institutions are located, the United States and Australia each had two institutions ranked among the top affiliations by document count.

**TABLE 2:** Most relevant affiliations.

Affiliation	Country	TP
Wuhan University	China	21
Chung-Ang University	South Korea	18
University of Technology Sydney	Australia	16
Information Technologies Institute	Greece	15
Texas A & M University	USA	15
Monash University	Australia	13
King Saud University	Saudi Arabia	12
Northumbria University	England	12
University of Iowa	USA	11
University of Twente	Netherlands	11

*Source:* Scopus n.d., Scopus preview - Scopus - Welcome to Scopus, viewed 14 May 2024, from https://www.scopus.com/home.uri

TP, total publications.

The spatial distribution of scientific production in AI research for disaster management revealed contributions from 76 countries worldwide, although African countries are underrepresented. Most authors were affiliated with institutions in China (533 documents), followed by the United States (485 documents), India (368 documents), Italy (146 documents), and the United Kingdom (138 documents). China and the United States were closely competing to become leaders in AI applications for disaster management, with their publication trends significantly surpassing those of other countries. It is important to acknowledge that there is an ongoing global race between China and the United States in the broader field of AI. Mnekhir (2023) has outlined the factors contributing to the significant advancements of both countries in AI technology development. The United States possesses key advantages in AI, such as advanced research capabilities, prestigious global academic institutions and large technology companies. Meanwhile, China has great investment potential, strong government incentives and well-established academic institutions.

Several other countries have also made significant progress in AI research for disaster management. Over the past decade, India has made significant contributions, with a sharp increase in publication growth beginning in 2022, closing the gap with China and the United States. Italy and the United Kingdom were among the earliest nations to publish AI research for disaster management, competing to lead in Europe.

### Citations

The AI publications on disaster management analysed in this study have been cited 14 165 times, averaging 16.70 citations per paper and 505.89 citations per year. The study calculated the average citations per year by dividing the total citations per article by the number of citation years. The findings indicate a significant increase in the average annual citations starting in 2015, consistently surpassing a rate of 2 citations per year. Newer publications tended to have higher average citations per year. The highest point occurred in 2018, with an average of 8.48 citations per year. This year also recorded the greatest total citations and the highest average citations per article, reaching 2732 and 59.39, respectively.

Table 3 shows the top 10 most cited studies according to the Scopus database. These studies have been cited more than 200 times, spread across different sources. Interestingly, four of the top five most cited papers were published in 2018, and the topics were related to flood hazards. The paper titled ‘Flood prediction using machine learning models: Literature review’ by Mosavi, Ozturk and Chau (2018) received the highest number of citations, with 825 total citations, far surpassing the other papers. The study presented an overview of ML models for flood prediction. The quality of short-term and long-term flood prediction using ML methods is considered to be improved by four key strategies, namely hybridisation, data decomposition, algorithm ensemble and model optimisation.

**TABLE 3:** Top 10 papers with the highest citations.

Title	Reference	Citation	Source title
Flood prediction using machine learning models: Literature review	Mosavi et al. (2018)	825	*Water*
A comparative assessment of decision trees algorithms for flash flood susceptibility modeling at Haraz watershed, northern Iran	Khosravi et al. (2018)	492	*Science of the Total Environment*
Flood susceptibility analysis and its verification using a novel ensemble support vector machine and frequency ratio method	Tehrany, Pradhan and Jebur (2015)	302	*Stochastic Environmental Research and Risk Assessment*
Survey of computational intelligence as basis to extensive flood management: Challenges, research directions, and future work	Fotovatikhah et al. (2018)	292	*Engineering Applications of Computational Fluid Mechanics*
Landslide susceptibility modeling applying machine learning methods: A case study from Longju in the Three Gorges Reservoir area, China	Zhou et al. (2018)	265	*Computers & Geosciences*
GIS-based modeling of rainfall-induced landslides using data mining-based functional trees classifier with AdaBoost, Bagging, and MultiBoost ensemble frameworks	Tien Bui et al. (2016)	248	*Environmental Earth Sciences*
A review on early forest fire detection systems using optical remote sensing	Barmpoutis et al. (2020)	235	*Sensors*
A multi-criteria optimisation model for humanitarian aid distribution	Vitoriano et al. (2011)	227	*Journal of Global Optimization*
Tackling climate change with machine learning	Rolnick et al. (2023)	204	*ACM Computing Surveys*
Hybrid artificial intelligence models based on a neuro-fuzzy system and metaheuristic optimization algorithms for spatial prediction of wildfire probability	Jaafari et al. (2019)	201	*Agricultural and Forest Meteorology*

*Source:* Scopus n.d., Scopus preview - Scopus - Welcome to Scopus, viewed 14 May 2024, from https://www.scopus.com/home.uri

In terms of the most cited countries, the United States held the top position with 3244 citations. Most of the top 10 highly cited countries were from Asia, including Iran (2442 citations), India (2121 citations), Vietnam (1703 citations), China (1685 citations), Malaysia (1431 citations), South Korea (1298 citations) and Hong Kong (1230 citations). Norway (1908 citations) and the United Kingdom (1571 citations) were among the most highly cited European countries in this field.

### Keyword analysis

Author keyword mapping was constructed by applying the full counting method. The minimum criterion for keyword occurrence was set at 4. Out of 2379 keywords, 73 met the threshold. The most frequently occurring keywords were ‘artificial intelligence’ (*n* = 171), followed by ‘disaster management’ (*n* = 137) and ‘machine learning’ (*n* = 104). Other notable keywords included ‘deep learning’, ‘decision support system’, ‘remote sensing’, ‘natural disasters’, ‘GIS’, ‘climate change’ and ‘natural disaster’.

**FIGURE 2:**
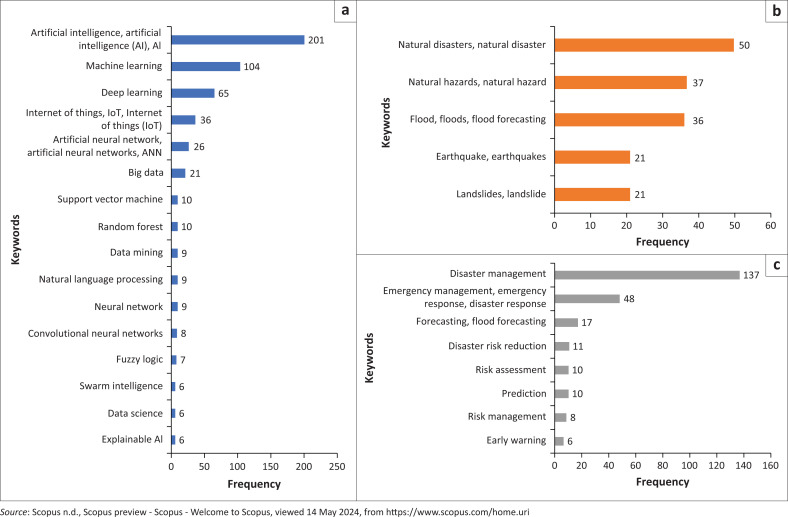
Number of keyword occurrences based on: (a) AI-related terminologies; (b) types of natural hazards; and (c) disaster management phases and activities.

This study grouped the keywords based on the types of AI-related terms, natural hazards, and disaster management phases and activities. When examining AI-related terms, ‘AI’ emerged as the most frequently used keyword with 201 occurrences (Figure 2). Machine learning and deep learning (DL) ranked second and third highest as top keywords, respectively. Various ML algorithms (including supervised, unsupervised and reinforcement learning models) have been extensively tested for different types of natural hazards. Nevertheless, the use of DL, a subset of ML, has been growing in disaster management research. This is because DL offers significant advantages over traditional ML methods, particularly in its ability to automatically learn and represent complex systems for prediction, detection or classification tasks (Linardos et al. 2022).

Artificial neural network (ANN) was the most commonly used AI method among author keywords. This aligns with Tan et al. (2021), who stated that ANN is the most widely applied AI algorithm in disaster management. However, certain AI methods require further exploration; for example, reinforcement learning and deep reinforcement learning have been rarely utilised in disaster mitigation studies (Sun et al. 2020). Additionally, the growing trend of adopting hybrid methods over single method should be highlighted, as they are considered to improve accuracy and efficiency in processing complex data.

The term ‘natural disaster(s)’ was a common keyword in disaster risk studies, but flooding was the most frequently studied specific type of hazard. Research on AI applications for flood disasters covered areas such as vulnerability and hazard modelling (Pradhan et al. 2023; Taromideh et al. 2024), prediction and forecasting (Mitra et al. 2016), early warning systems (Mohd Zain & Ithnin 2022; Nguyen et al. 2020), risk assessment (Pham et al. 2021), event characteristic estimation (Nair & Rao 2017; Vallimeena et al. 2018), inundation detection (Peter, Matjaž & Krištof 2013) and response operations (Nasim & Ramaraju 2019). Other widely studied natural hazards included earthquakes and landslides. These findings are consistent with the systematic review by Tan et al. (2021), which identified floods, landslides and earthquakes as some of the most extensively studied natural hazards in AI model applications. Artificial intelligence models have, in fact, been applied to other natural hazards (e.g. droughts, wildfires, storms and tsunamis), although in limited numbers. This further highlights a gap in AI applications for studies concentrating on these specific hazard types.

Regarding disaster management phases and activities, ‘disaster management’ was frequently used as a keyword. However, this does not necessarily imply that the studies provided a comprehensive analysis of disaster management, which encompasses mitigation, preparedness, emergency response and recovery. Only a small portion of research truly explored the application of AI across the entire disaster management cycle in a holistic manner. For instance, Keskin et al. (2018) introduced the Disaster Management and Decision Support System (AYDES), an integrated platform for disaster and emergency data, reports, statistics, task monitoring, queries, analysis and more from pre-disaster to post-disaster stages. Cicek and Kantarci (2023) conducted a comprehensive study on the role of mobile crowdsensing throughout all phases of disaster management.

This study identified emergency response as the most frequently occurring keyword when examining disaster management phases in a partial context. As noted by Sun et al. (2020), the majority of AI applications currently focus on disaster response. Given the global commitment to prioritising a risk reduction approach, future AI utilisation should ideally place greater emphasis on mitigation and preparedness phases, while still recognising the importance of response and recovery efforts. With ongoing advancements in AI models and the growing availability of real-time data, there is an increasing opportunity to enhance AI’s role in minimising fatalities and losses from natural hazard events.

**FIGURE 3:**
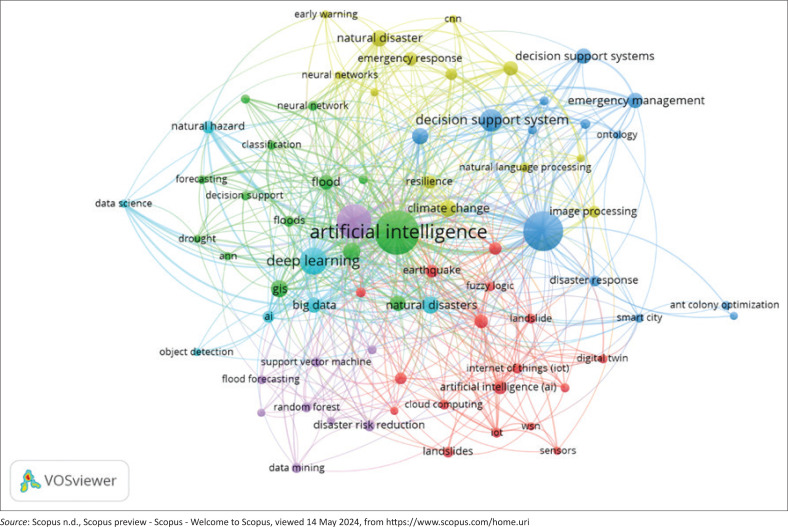
Network visualisation of author keywords (Weights: Occurrences).

Network visualisation analysis can help scholars identify relationships between keywords (represented by nodes) and analyse the connections among these nodes. The size of the circle indicates the number of documents that use the keyword, while keyword clustering is indicated by different colours. The findings revealed that six clusters were formed from keyword mapping (Figure 3).

Cluster I (red) focused on disaster monitoring and prediction using Internet of Things (IoT) devices, with wireless sensor networks (WSNs) crucial for IoT operations. Wireless sensor networks identify and track devices to gather information from interconnected sensors (Landaluce et al. 2020). Sensors are vital in integrating data through wireless technology, commonly used in IoT applications (Jamshed et al. 2022). Artificial intelligence enhances the reach, distribution and localisation of WSNs, benefiting various fields (Osamy et al. 2022). For instance, Vunabandi et al. (2015) developed a flood early warning system using a WSN-based siren with components like Arduino and solar panels. Boulouard et al. (2022) combined AI and IoT for real-time flood monitoring, where sensors relay data to a local centre using the low-range wide area network protocol for flood prediction.

Cluster II (green) examined AI-based geospatial technology for risk management, utilising geographic information systems (GIS) to process and analyse location-based data, and remote sensing to gather earth surface information without direct contact. Artificial intelligence enhances the integration of GIS and remote sensing, producing accurate vulnerability and disaster risk management models and providing faster and better damage assessments than traditional methods (Ivić 2019). Geospatial Artificial Intelligence (GeoAI) has emerged as a crucial interdisciplinary field, integrating AI with spatial science methods to address spatial-related issues, including disaster risk assessment (Rezvani et al. 2024).

Cluster III (dark blue) covered the use of decision support systems (DSS) in disaster emergency management. Decision support systems integrate information systems and technology to aid decision-making (Yun, Ma & Yang 2021). Artificial intelligence integration in DSS improves decision-making accuracy in uncertain environments (Gupta et al. 2022). Vargas Florez et al. (2015) developed an AI-based DSS model for effective humanitarian aid during emergencies. Nasar et al. (2023) highlighted the benefits of DSS and AI in search and rescue operations. While DSS is emphasised for response activities, it also plays a crucial role in risk reduction measures, including structural planning, nature-based solutions, financial tools, education and administration (Newman et al. 2017).

Cluster IV (yellow) addressed social media analysis for emergency response, where social media is a key tool for communication during emergencies. Herfort et al. (2014) developed a method to extract social media data to improve situational awareness during floods. Powers et al. (2023) used ML to analyse tweets during Hurricane Harvey for first responders. Nunavath and Goodwin (2018) reviewed AI applications for analysing social media data in disaster management, focusing on text and image classification.

Cluster V (purple) highlighted the application of ML algorithms for disaster risk reduction, emphasising pre-disaster activities to build capacity and minimise risks (Sreelakshmi & Chandra 2022). Various ML techniques were employed to predict disaster events and map hazards with high accuracy (Band et al. 2020; Saleem & Rashid 2023; Swain et al. 2023; Zhou et al. 2018). Recent advancements in ensemble and hybrid models have shown superior performance compared to single-method approaches (Mosavi & Ardabili 2023).

Cluster VI (light blue) emphasised the use of big data and DL for disaster management. Big data is characterised by high volume, velocity and variety, which are key in producing accurate disaster forecasts. Liu et al. (2023) highlighted the promise of big data and AI in improving typhoon risk forecasting. Deep learning is crucial in automating analytical models and solving problems (Janiesch, Zschech & Heinrich 2021), advancing fields like speech processing and computer vision (Dong, Wang & Abbas 2021). Deep learning has enabled AI to perform complex cognitive tasks at human-like levels (Perconti & Plebe 2020).

**FIGURE 4:**
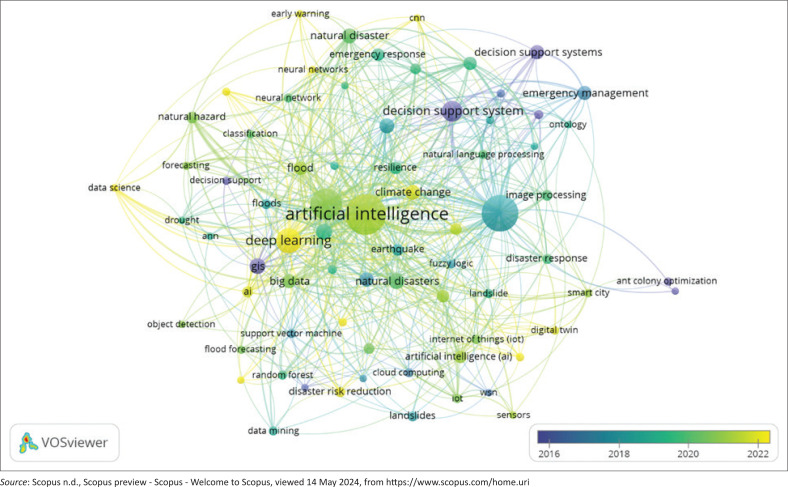
Overlay visualisation of author keywords (Weights: Occurrences; Score: Average Publication per Year).

Research developments can be examined from the trend of keyword usage over time (Figure 4). In this study, the overlay visualisation applied colours to keywords based on their average time of occurrence. The blue circle indicates terms with an earlier average time of occurrence, while the yellow circle represents terms that appeared more recently. Keywords with older average annual publications included ant colony optimisation, swarm intelligence, DSS, computational intelligence, WSN, GIS, risk assessment and risk management. Meanwhile, keywords with newer average annual publications included data science, DL, convolutional neural networks, explainable AI, edge computing, climate change, early warning and disaster risk reduction.

## Conclusion

Natural hazard risks are increasingly concerning because of the growing number of exposed elements, global environmental changes and accelerated land degradation. The advancement of AI methods offers significant opportunities to enhance disaster management throughout its entire cycle. A bibliometric analysis revealed rapid literature growth over the last decade, with 2023 seeing the highest number of publications at 151 documents. The top contributing source was the International Archives of the Photogrammetry, Remote Sensing and Spatial Information Sciences – ISPRS Archives (22 documents), and the most prolific author was Amir Mosavi (10 documents).

China led in producing authors who research AI applications for disaster management, with Wuhan University being the most prominent affiliation. However, the United States was the most cited country. The most cited article was ‘Flood prediction using machine learning models: Literature review’ by Mosavi et al. (2018). Bibliometric exploration of author keywords identified six research hotspot clusters:

1. Disaster monitoring and prediction with IoT device networks.
2. Utilisation of AI-based geospatial technology for risk management.
3. Utilisation of DSS for disaster emergency management.
4. Social media analysis for emergency response.
5. Application of ML algorithms to support disaster risk reduction.
6. Utilisation of big data and DL for disaster management.

Artificial intelligence model development holds promising prospects for helping disaster management stakeholders make quick, accurate and cost-effective decisions. However, there is still great potential to explore AI tools in areas that have received less attention. Practical challenges, such as data, resources, model complexity, ethical implications and compatibility, remain. It is also important to mention that this work has not examined whether the effectiveness of AI-based approaches proposed by existing published research has been tested through empirical evidence. This highlights the necessity for further research to bridge the gap between the theoretical potential of AI and its real-world benefits in disaster management practices.
